# A Protective and Safe Intranasal RSV Vaccine Based on a Recombinant Prefusion-Like Form of the F Protein Bound to Bacterium-Like Particles

**DOI:** 10.1371/journal.pone.0071072

**Published:** 2013-08-12

**Authors:** Alan Rigter, Ivy Widjaja, Hanneke Versantvoort, Frank E. J. Coenjaerts, Maarten van Roosmalen, Kees Leenhouts, Peter J. M. Rottier, Bert Jan Haijema, Cornelis A. M. de Haan

**Affiliations:** 1 Virology Division, Department of Infectious Diseases & Immunology, Faculty of Veterinary Medicine, Utrecht University, Utrecht, The Netherlands; 2 Mucosis B.V., Groningen, The Netherlands; 3 Department of Medical Microbiology, University Medical Center Utrecht, Utrecht, The Netherlands; University of Georgia, United States of America

## Abstract

Respiratory syncytial virus (RSV) is an important cause of respiratory tract disease in infants and the elderly. Currently, no licensed vaccine against RSV is available. Here we describe the development of a safe and effective intranasal subunit vaccine that is based on recombinant fusion (F) protein bound to the surface of immunostimulatory bacterium-like particles (BLPs) derived from the food-grade bacterium *Lactococcus lactis*. Different variants of F were analyzed with respect to their conformation and reactivity with neutralizing antibodies, assuming that F proteins mimicking the metastable prefusion form of RSV F expose a more extensive and relevant epitope repertoire than F proteins corresponding to the postfusion structure. Our results indicate that the recombinant soluble ectodomain of RSV F readily adopts a postfusion conformation, generation of which cannot be prevented by C-terminal addition of a trimerization motif, but whose formation is prevented by mutation of the two furin cleavage sites in F. While the putative postfusion form of F is recognized well by the monoclonal antibody Palivizumab, this is much less so for the more potently neutralizing, prefusion-specific antibodies D25 and AM22. Both addition of the trimerization motif and mutation of the furin cleavage sites increased the reactivity of F with D25 and AM22, with the highest reactivity being observed for F proteins in which both these features were combined. Intranasal vaccination of mice or cotton rats with BLPs loaded with this latter prefusion-like F protein (BLP-F), resulted in the potent induction of F-specific immunoglobulins and in significantly decreased virus titers in the lungs upon RSV challenge. Moreover, and in contrast to animals vaccinated with formalin-inactivated RSV, animals that received BLP-F exhibited high levels of F-specific secretory IgA in the nose and RSV-neutralizing antibodies in sera, but did not show symptoms of enhanced disease after challenge with RSV.

## Introduction

Human respiratory syncytial virus (RSV) causes acute upper and lower respiratory tract infections and is a major cause for hospitalization of infants in the first year of life. Furthermore, re-infection occurs frequently and sterilizing immunity is never firmly established. RSV also causes a significant disease burden and mortality in the elderly, comparable to influenza. Currently, the only available option to prevent RSV-mediated disease is the passive administration of the commercially available RSV-neutralizing monoclonal antibody (MAb) Palivizumab. However, its use is restricted to infants considered at high risk of developing severe respiratory disease due to its high costs. Although there is a need for a vaccine to protect specific risk groups or population at large, there is currently no licensed vaccine against RSV available (for recent reviews on RSV see [1], [2], [3], [4].

RSV vaccine development has been hampered by the disastrous results obtained with the formalin-inactivated (FI) virus vaccine that was tested in the 1960s (reviewed by [4]). Disease severity and hospital admission rates were increased in vaccinated children, who were naturally infected with RSV post vaccination, and several deaths occurred. The mechanism of this vaccine-induced disease enhancement remains incompletely understood, but appears associated with a Th2-skewed immune response, high levels of non-neutralizing antibodies, absence or low levels of neutralizing antibodies and recruitment of eosinophils to lung epithelia. Nevertheless, a large number of RSV vaccine strategies have been explored with varying success, including live-attenuated RSV strains, subunit vaccines and viral vectored vaccines [3], [4]. Obviously, a successful RSV vaccine should induce protective immunity, but no immunopathology.

RSV is an enveloped, negative-strand RNA virus belonging to the family *Paramyxoviridae*. The envelope contains two major glycoproteins, which are the main targets of neutralizing antibodies: the attachment protein G and the fusion protein F (reviewed by [1]). There are two RSV antigenic groups (A and B), which differ more in their G than F proteins. The F protein appears to be a more efficient neutralizing and protective antigen compared to G [5]. This may be related among others to the high carbohydrate content of the G protein, which may shield the protein from immune recognition [6], [7]. In addition, the G protein is also secreted from infected cells [8], [9], in which form it may function as an antigen decoy [10]. The F protein not only functions to fuse viral and host membranes 11,12, but also plays a major role in virus-cell attachment [13], [14], [15]. Neutralizing antibodies targeting F may therefore interfere with virus-cell attachment and/or with virus-cell fusion. The RSV neutralizing MAb Palivizumab that is used as prophylaxis for RSV infection recognizes a highly conserved epitope in the F protein [2], [16].

The RSV F protein is a type I membrane protein that is synthesized as an inactive precursor protein (F0) that assembles into trimers (reviewed by [1]). This precursor protein is cleaved by furin-like proteases into F2, p27 and F1 during its transport through the secretory route [17], [18]. Homotrimers of F2 and F1, which are covalently linked via disulfide bridges, form the metastable prefusion structure [11], [12]. The F1 part contains heptad repeats A and B (HRA and HRB), the fusion peptide and the transmembrane domain, the latter two positioned at opposite sides of the molecule. Upon virus-cell attachment, conformational changes in the RSV F protein lead to the insertion of the hydrophobic fusion peptide into a host cell membrane. This fusion intermediate then refolds into the highly stable postfusion structure. The assembly of the postfusion structure is dictated by the formation of a six-helix bundle (6HB). This 6HB contains the HRA and HRB region of each monomer in an antiparallel orientation, as a result of which the transmembrane domain, located downstream of HRB, and the fusion peptide, located upstream of HRA, are positioned in adjacent positions consistent with fusion of the viral and host membranes having been achieved. The structure of the F protein in its postfusion conformation has recently been elucidated [19], [20].

The immune-enhancing properties of adjuvants are normally used to overcome the low immunogenicity of recombinant protein vaccines. In the current study, however, we made use of innovative immunostimulatory carriers that consist of peptidoglycan spheres produced from the food-grade bacterium *Lactococcus lactis* [21]. The bacterium is killed at low pH and high temperature, which generates bacterium-like particles (BLPs) that are non-living and deprived of intact surface proteins and intracellular content. The BLPs activate antigen presenting cells of the innate immune system by Toll-like receptor 2 (TLR-2) interaction [22], and were shown to act as potent immunostimulants for intranasal immunization. The BLPs can be admixed with a vaccine antigen [23], [24], but may also be loaded with antigen that is genetically fused to a peptidoglycan binding moiety. Thus, BLPs carrying Malaria parasite, *Yersinia pestis* and pneumococcal antigens bound to BLPs were shown to induce robust and protective immune responses in mouse models [22], [25], [26], [27], [28].

Using the BLP technology we aim to develop a needle-free, mucosal vaccine that protects against RSV using the F protein as our antigen of choice. To this end we expressed recombinant soluble variants of the F protein fused to a peptidoglycan binding moiety, which were subsequently loaded onto the BLPs (BLP-F). Different variants of F were analyzed with respect to their conformation and reactivity with neutralizing antibodies, assuming that F protein mimicking the metastable prefusion form of RSV F exposes a more extensive and relevant epitope repertoire than F proteins corresponding to the stable inactive postfusion structure. In agreement with this assumption, Magro and coworkers recently showed that antibodies specific for the prefusion form of F account for most of the neutralizing activity found in human sera [29]. Our results indicate that intranasal vaccination of mice or cotton rats with BLPs displaying F proteins that expose prefusion-specific epitopes induces strong F-specific IgG responses in sera that have potent RSV neutralizing capacity. Furthermore, such a vaccine resulted in substantial secretory IgA (SIgA) levels in the nose, while virus titers in the lungs were significantly decreased after challenge compared to mock-vaccinated animals. In contrast to animals that received FI-RSV, no signs of vaccination-induced enhanced disease symptoms could be observed upon RSV challenge after intranasal vaccination with BLP-F preparations.

## Materials and Methods

### Ethics Statement

Animal experiments and study protocols were either approved by the Committee for Animal Experimentation of the University of Groningen (the Netherlands) or by Sigmovir Institutional Animal Care Utilization Committee. The study was carried out in strict accordance with the guidelines provided by the Dutch Animal Protection Act or National Institutes of Health, respectively. All efforts were made to minimize suffering.

### Genes and Expression Vectors

**Figure 1:**
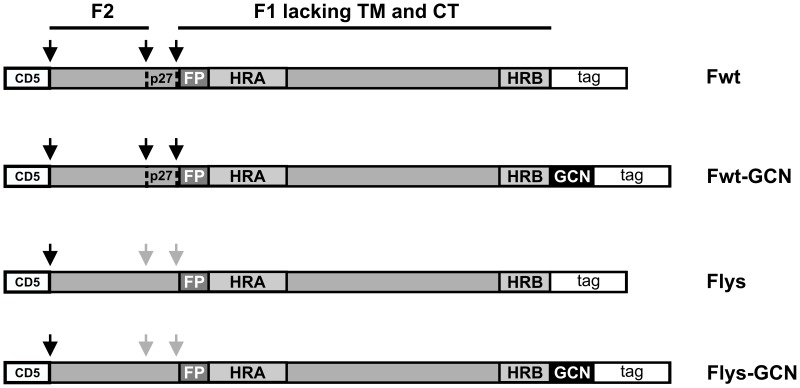
Schematic representation of the different recombinant soluble RSV F protein constructs. RSV F proteins lacking the transmembrane domain (TM) and cytoplasmic tail (CT) were genetically fused to a CD5 signal peptide (CD5) and to a carboxy-terminal tag (tag). When indicated a GCN4 trimerization motif (GCN) was introduced between the F protein and the tag. The tag either consisted of a triple Strep-tagII, a LysM peptidoglycan binding domain, or of a combination of the two (Fig. S1). The F2 and F1 subunits of F are indicated, as well as the p27 peptide (P27) that is released after furin cleavage. Protease cleavage sites are indicated by black arrows. Grey arrows indicate mutated furin cleavage sites. The approximate location of the fusion peptide (FP), heptad repeat A (HRA) and B (HRB) is also shown.

Two variants of a cDNA clone corresponding to residues 26 to 515 of the F protein of an European isolate of RSV serotype A (Genbank accession number JX015498.1) [30] were synthesized using human-preferred codons by GenScript USA Inc. While one cDNA clone encodes the wild-type F protein ectodomain, the other clone encodes a F protein ectodomain in which the arginine residues in the two multibasic furin cleavage sites are mutated into lysines (RARR to KAKK and KKRKRR to KKKKKK). Each cDNA was cloned into the pCD5 expression vector for efficient expression in mammalian cells [31], [32]. The pCD5 vector had been modified such that the F protein-encoding sequences were cloned in frame downstream of a DNA sequence coding for a CD5 signal peptide and when indicated upstream of sequences encoding an artificial GCN4 isoleucine zipper trimerization motif [33] and a tag (see Fig. 1). The tag either consisted of a triple Strep-tagII (IBA, Germany), a LysM peptidoglycan binding domain [21], or a LysM domain followed by a triple Strep-tagII. For a schematic representation of the different expression constructs see Fig. 1, while the sequences of the GCN4 motif and the tags used is provided in Fig. S1. Codon-optimized DNA fragments encoding the variable heavy and light chains of antibody D25 [34] and AM22 [35] were synthesized by GenScript USA Inc and cloned in-frame into pCAGGS mammalian expression vectors containing human IgG1 heavy and light constant domains, respectively.

### Expression and Purification of Recombinant Proteins

pCD5 expression vectors containing RSV F ectodomain-encoding sequences were transfected into HEK293T cells using polyethyleneimine I (PEI) in a 1∶5 w/w ratio (DNA/PEI). At 6 h post transfection, the transfection mixture was replaced by 293 SFMII expression medium (Invitrogen), supplemented with sodium bicarbonate (3.7 g/liter), glucose (2.0 g/liter), Primatone RL-UF (3.0 g/liter), penicillin (100 units/ml), Streptomycin (100 µg/ml), glutaMAX (Gibco), and 1.5% dimethyl sulfoxide. Tissue culture supernatants were harvested 5–6 days post transfection. F proteins were either purified using Strep-tactin Sepharose beads according to the manufacturer’s instructions (IBA, Germany) for further analysis of the protein or bound to BLPs as described below. The concentration of Strep-tactin purified protein was determined by using a Nanodrop 1000 spectrophotometer (Isogen Life Sciences) according to the manufacturer’s instructions. The D25 or AM22 expression vectors encoding the heavy and light chains were cotransfected at a 1∶1 ratio into HEK293T cells similarly as described above. Antibodies secreted in the cell culture supernatant were bound to protein A sepharose beads (GE Healthcare), after which they were eluted using 0.1 M citric acid pH 3.0. The eluates were immediately neutralized using 1 M Tris-HCl pH 8.8.

### Gel Electrophoresis of Recombinant Proteins

Expression and secretion of recombinant proteins was confirmed by sodium dodecylsulfate (SDS)-polyacrylamide (PA) gel electrophoresis (SDS-PAGE; 10% NuPAGE BisTris, Invitrogen) followed by Western blotting using anti-Strep-tag antibody conjugated with horse radish peroxidase (HRP) (StrepMAB-classic-HRP, IBA), Palivizumab (Synagis, Abbott Laboratories) followed by HRP-conjugated anti-human IgG antibody (ITK Southern Biotech). This latter antibody was also used to confirm expression of recombinant antibodies AM22 and D25. Prior to SDS-PAGE analysis, the samples were resuspended in Laemmli sample buffer (LSB) that either did or did not contain 5% 2-mercaptoethanol (ME; Sigma), and heated at 96°C for 5–15 minutes when indicated.

### Limited Proteolysis

Purified F proteins (2 µg) were (mock-)treated with varying amounts of TPCK treated trypsin (0.04–0.005 µg range per 10 µl reaction volume) from bovine pancreas (Sigma) for 30 min at 23°C. Subsequently, the samples were put on ice and trypsin inhibitor (Sigma) was added, after which they were analyzed by SDS-PAGE as described above. Protein bands were visualized by using a Colloidal Blue Staining kit (Invitrogen).

### Enzyme-linked Immunosorbent Assay (ELISA) Analysis of Recombinant Soluble F

96-well Nunc maxisorp plates were coated with different F protein preparations (50 ng/well) o/n at 4°C. After blocking (phosphate buffered saline [PBS] with 0.1% Tween-20 v/v and 3% bovine serum albumin w/v) and extensive washing (PBS with 0.05% Tween-20 v/v), the plates were incubated with limiting dilutions of Palivizumab (starting with a 1 in 8000 dilution of a 3 mg/ml stock), AM22 (starting with a 200-fold dilution of a 0.7 mg/ml stock) or D25 (starting with a 200-fold dilution of a 1 mg/ml stock). After extensive washing, the plates were incubated with HRP conjugated goat-anti-human IgG antibodies (ITK Southern Biotech) at a 1∶500 dilution for one hour at room temperature. Detection of HRP reactivity was performed using tetramethylbenzidine substrate (BioFX) and a ELISA plate reader (EL-808 from Biotek). All experiments were repeated 2–3 times. The results of representative experiments are shown.

### Binding of F to BLPs

BLPs, previously termed GEM particles, were prepared as described earlier [21]. In brief, cells of an overnight culture of *L. lactis* strain MG1363*acmAΔ1* were harvested and washed once with sterile distilled water. The cells were resuspended in 10% trichloroacetic acid and placed in a hot water bath of 99°C for 30 min. The acid and heat treatment kills the bacteria and generates the so-called BLPs. After acid and heat treatment the BLPs were pelleted and washed three times in (PBSand finally resuspended in PBS and stored at −80°C. One mg of BLP was defined as approximately 8×10^9^ nonliving particles. Preparation of BLP-based vaccines is also described in detail elsewhere [21]. In short, culture supernatants containing the fusion recombinant proteins were concentrated with a Vivaspin (Vivaproducts). Binding of antigens was achieved by mixing the concentrates with BLP particles under gentle agitation for 30 min at room temperature, followed by extensive washing with PBS to remove unbound proteins. The amount of F protein bound to the BLPs was determined by comparative Colloidal Blue staining (Invitrogen) of F proteins in SDS-PA gel relative to bovine serum albumin (BSA) protein standards. BLPs carrying Flys-GCN protein (BLP-F) were analyzed by immunofluorescence microscopy using Palivizumab and FITC-labeled goat-anti-human secondary antibodies (Southern Biotech, USA). Prior to each animal experiment BLP-F vaccine preparations were assembled, analyzed, and stored at 4°C as single use aliquots.

### Immunization of Mice

Balb/c mice (6–8 weeks) purchased from Harlan (Zeist, The Netherlands) were divided in 3 groups (N = 8 per group). All animals were immunized with prime vaccination on day 0, a booster vaccination on day 10, and a second booster on day 20. Group 1 and 2 were vaccinated intranasally with either 10 µg Flys-GCN with a C terminal LysM domain bound to 0.3 mg BLPs (10 µl per dose) or with 10 ug of a similar F protein (Flys-GCN with a C terminal LysM domain and a ST3 tag) in the absence of BLPs, respectively. Group 3 received formaldehyde inactivated RSV (FI-RSV) (1/100 diluted, 50 µl per dose; prepared according to [36]) in both their calf muscles equally divided over both injection sites. 4 weeks after the second booster vaccination, blood samples were drawn by orbital puncture. Sera were obtained by centrifugation of blood at 1,200×g for 5 min and the samples were subsequently stored at −20°C until further analysis. Nasal washes were obtained by flushing the nasopharynx with 1 ml PBS with protease inhibitor (Roche diagnostics). The lavage fluid was centrifuged for removal of cells and stored at −20°C until further use.

### Challenge Virus

The prototype Long strain of RSV (ATCC, Manassas, VA) was propagated in HEp-2 cells after serial plaque-purification to reduce defective-interfering particles. A pool of virus designated as RSV/A/Long in sucrose stabilizing media (Sigmovir Biosystems, Inc, USA) was used for the in vivo experiments. The stock of virus is stored at −80°C and has been characterized *in vivo* using the cotton rat model for replication of RSV in the upper and lower respiratory tract.

### Immunization of Mice Followed by RSV Challenge

One mouse vaccination-challenge experiment was carried out at Sigmovir Biosystems, Inc, USA. Eighteen Balb/c mice (6–8 weeks) were purchased from Harlan (USA) and maintained and handled under veterinary supervision. Animals of all 3 groups (N = 6 per group) were immunized with prime vaccination on day 0, a booster vaccination on day 14, and a second booster on day 28. Group 1 and 2 were (mock-)vaccinated intranasally with either PBS (group 1, mock; 10 µl per dose), or BLP-F (group 2, 10 µg Flys-GCN bound to 0.5 mg of BLP per 10 µl dose). Group 3 received FI-RSV (1/100 diluted, 50 µl per dose; prepared according to [36]) in both their calf muscles, equally divided over both injection sites. Fourteen days after the last vaccination all animals were challenged intranasally with 100 µl of RSV suspension (10^6^ plaque forming units [pfu] RSV/A/long per animal). Immunizations and challenge were performed under isoflurane anesthesia. Blood samples were drawn on day 0, 14, 28 and 48 by orbital puncture. Animals were sacrificed by CO_2_ inhalation five days after challenge and the lungs were removed from the thorax. The right lung was inflated with formalin for histopathology (see below), while the lingular lobe of the left lung was homogenized and used for virus titrations (see below).

### Immunization of Cotton Rats Followed by RSV challenge

One cotton rat vaccination-challenge experiment was carried out at Sigmovir Biosystems, Inc, USA. Fifteen inbred female Sigmodon hispidus cotton rats between 6 to 8 weeks of age (Sigmovir Biosystems, Inc., Rockville MD) were maintained and handled under veterinary supervision. Animals of all 3 groups (N = 5 per group) were immunized with prime vaccination on day 0, a booster vaccination on day 14, and a second booster on day 28. Group 1 and 2 were (mock-)vaccinated intranasally with either PBS (group 1, mock; 25 µl per dose), or BLP-F (25 µg Flys-GCN bound to 1.25 mg of BLP per 25 µl dose). Group 3 received FI-RSV (1/100 diluted, 50 µl per dose; prepared according to [36] in both their quadriceps equally divided over both injection sites. Fourteen days after the last vaccination all animals were challenged intranasally with 100 µl of RSV suspension (10^5^ pfu RSV/A/long virus per animal). Immunizations and challenge were performed under isoflurane anesthesia. Blood samples were drawn on day 0, 14, 28 and 48 by orbital puncture. Animals were sacrificed by CO_2_ inhalation five days after challenge and the lungs were removed from the thorax. The right lung was inflated with formalin for histopathology (see below), while the lingular lobe of the left lung was homogenized and used for virus titrations (see below).

### Analysis of the Antibody Response Against RSV F using ELISA

The antibody response to RSV F was determined using ELISA, similarly as described previously [23]. Briefly, ELISA plates with high binding capacity (Greiner) were coated overnight at 4°C with Strep-tactin-purified Fwt (Fig. 1). The plates were blocked with blocking buffer (50 mM carbonate-bicarbonate pH 9.7 with 2,5% Protifar® plus w/w [Nutricia, the Netherlands]) and subsequently washed. For determination of total serum IgG, specific IgG2A or IgG1 titers, samples were applied to the plates in serial triple-fold dilutions using a multichannel pipette. A mouse control serum sample, positive for RSV F was included in each plate. Mouse IgG (Sigma) diluted in triplicate (first well 0.5 µg/ml) was used to generate a calibration curve. The plates were incubated for 1.5 hrs at 4°C, followed by 3 washing steps. The plates were subsequently incubated with the appropriate conjugate (anti-mouse IgG, IgG1 or IgG2A conjugated with HRP, Southern Biotech) for 1 hr at 4°C. HRP activity was detected similarly as described above. The anti-F IgG antibody levels in serum (expressed in µg/ml) were determined using the calibration curve (parameters of curve determined by 4-parameterfit). For detection of SIgA in nose lavages, a similar method was employed, using undiluted washes (and dilutions thereof) for the detection of antibodies in ELISA. The plates were incubated with the appropriate conjugate (anti-mouse IgA-HRP conjugate; Southern Biotech, USA). Titers reported are the reciprocal of the calculated sample dilution corresponding with an OD of at least 0.3 after background correction.

### RSV Neutralizing Antibody Assay

The RSV neutralizing antibody titers in the serum samples were determined by analyzing the reduction of RSV infectivity in the presence of serially diluted serum samples. Two different methods were employed, First, heat inactivated serum samples were diluted 1∶10 with EMEM and serially diluted further 1∶4. Diluted serum samples were incubated with RSV/A/Long (25–50 pfu) for 1 hour at room temperature and inoculated in duplicates onto confluent HEp-2 monolayers in 24-well plates. After one hour incubation at 37°C in a 5% CO_2_ incubator, cells were overlayed with 0.75% methylcellulose medium and plates were incubated at 37°C in the incubator. Four days later the overlay was removed and the cells were fixed with 0.1% crystal violet stain for one hour, then rinsed, and air dried. The corresponding reciprocal neutralizing antibody titers were determined at the 60% reduction end-point of the virus control using the statistics program “plqrd.manual.entry”. The geometric means and standard error for animals in a group at a given time were calculated.

Alternatively, heat inactivated serum samples or MAbs were diluted 1∶10 with DMEM supplemented with 2% fetal calf serum (Bodinco B. V.), 100 U/ml Penicillin and 100 µg/ml Streptomycin and serially diluted further 1∶2. Diluted serum samples were incubated with RSV/A/Long (500 50% tissue culture infectious doses [TCID50]) for 1 hour at room temperature and inoculated in duplicates onto confluent HEp-2 monolayers in 96-well plates. After 30 hours incubation at 37°C in a 5% CO_2_ incubator, cells were fixed with 3.7% formaldehyde solution and stained with Palivizumab and a goat-anti-human IgG conjugated with Dylight488 (Jackson Immunoresearch). Images of cells in each well were taken using an EVOS fluorescence microscope (Life Science Technologies) and the amount of infection was calculated by determining the total area of infected cells using ImageJ. The neutralizing titers were determined at the 50% reduction point of the virus control using logarithmic equation derived by plotting dilution versus percentage of infection.

### Determination of Virus Titers in the Lung

Lung homogenates were clarified by centrifugation and diluted 1∶10 and 1∶100 in EMEM. Confluent HEp-2 monolayers were infected in duplicates with 50 µl per well starting with undiluted (neat) samples followed by diluted homogenates in 24-well plates. After one hour incubation at 37°C in a 5% CO_2_ incubator, the inocula were removed and wells were overlayed with 0.75% methylcellulose medium and plates restored into the 37°C incubator. After 4 days of incubation the overlay was removed and the cells were fixed with 0.1% crystal violet stain for one hour, then rinsed, and air dried. Plaques were counted and virus titers are expressed as pfu per gram of tissue. Viral titers were calculated as the geometric mean plus standard error for all animals in that group at a given time.

### Histopathology

Lung were dissected and inflated with 10% neutral buffered formalin to their normal volume, and then immersed in the same fixative solution. Following fixation, the lungs were embedded in paraffin, sectioned and stained with hematoxylin and eosin. Two parameters of pulmonary inflammation were evaluated: interstitial pneumonia, defined as inflammatory cell infiltration and thickening of alveolar walls, and alveolitis, defined as cells within the alveolar spaces. Slides were scored blind on a 0–4 severity scale. The scores were subsequently converted to a 0–100% histopathology scale.

### Statistical Analysis

Immunogenicity and viral load analyses were conducted using the per protocol (PP) population, defined as all subjects who received the full amount of treatments and had blood drawn for serology testing on indicated days. Mean titers (MTs) and geometric mean titers (GMTs), were computed and compared based on a Mann-Whitney U test. The difference between BLP-F and the other groups present in the experiment was assessed.

## Results

### Design of RSV F Constructs

In the present study, we aimed to develop an RSV vaccine based on recombinant soluble F protein ectodomains bound to the surface of the immunostimulatory BLPs. Previously, others have shown that expression of fully cleaved, soluble F protein gives rise to multimeric forms that are either in the postfusion conformation [18], [37], [38] or in a pre-triggered state that easily adopts a postfusion conformation upon exposure to low-molarity buffer [39]. In similar preparations that were used for structure determination the F protein was also observed in its postfusion conformation [19], [20]. However, inhibition of the furin-cleavage of F appears to prevent the protein from adopting the postfusion conformation [37], [38], consistent with cleavage of F being required for activation of membrane fusion [18].

We and others have previously demonstrated that recombinant soluble class I fusion proteins can be stably maintained in their prefusion conformation by the C-terminal addition of an artificial trimerization domain [32], [40], [41], [42]. In the present study, we applied a design similar to what we previously used to produce recombinant soluble bioactive influenza A virus hemagglutinin protein [32], [43], [44], for the expression of soluble RSV F protein. Thus, the human codon-optimized RSV F ectodomain sequence of an European type A clinical isolate [30] was preceded by a signal peptide-encoding sequence and followed by sequences coding for the GCN4 isoleucine zipper trimerization motif [33] and the appropriate tags (referred to as Fwt-GCN; Fig. 1). For comparison, the F protein was also cloned into vectors without sequences encoding the trimerization motif (referred to as Fwt). The tags consisted either of a triple Strep-tag (ST3) for easy purification, a LysM peptidoglycan binding domain [21] for binding of the F proteins to BLPs, or a combination thereof as it turned out that, for yet unknown reasons, GCN4-containing F constructs were expressed to higher levels when also the LysM domain was present (data not shown). In addition to the “wild-type” constructs (Fwt and Fwt-GCN) that specified F proteins with non-modified furin cleavage sites, we also constructed expression vectors encoding F proteins with modified furin-cleavage sites, in which all arginines of the multibasic furin cleavage sites were substituted by lysines (Flys and Flys-GCN).

### Expression and Characterization of Recombinant Soluble RSV F Proteins

**Figure 2:**
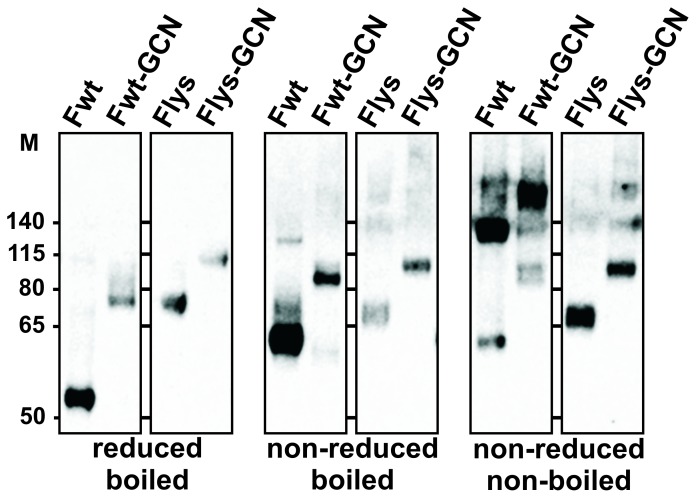
Western blot analysis of recombinant F proteins. Purified F proteins (Fwt, Fwt-GCN, Flys and Flys-GCN) were analyzed by SDS-PAGE followed by Western blotting. Fwt-GCN and Flys-GCN contain a C-terminal LysM domain and ST3 tag. Fwt and Flys contain a C-terminal ST3 tag. The presence (reduced) or absence (non-reduced) of 2-mercaptoethanol in the sample buffer and boiling of the samples prior to electrophoresis is indicated. F proteins were detected using Palivizumab. The size of the molecular mass markers (in kDa) is shown on the left (M), while position of the markers in the gels is indicated.

Expression of the F protein ectodomains was achieved by transient transfection of the expression plasmids into HEK293T cells. F proteins expressed and secreted into the cell culture media were purified by a single step protocol using Strep-tactin beads and analyzed by SDS-PAGE followed by Western blotting using Palivizumab as shown in Fig. 2. The epitope recognized by Palivizumab is located in the F1 part of the F protein (Fig. 1). When the F ectodomains were subjected to SDS-PAGE under reducing conditions (i.e. in the presence of 2-mercaptoethanol), which results in separation of the otherwise disulfide-linked F1 and F2 moieties, the F1 proteins migrated with different electrophoretic mobilities corresponding to the absence or presence of the GCN4-LysM sequence (Fig. 2). Furthermore, the F proteins migrated at a higher position in the gel when the furin-cleavage sites had been mutated (compare Fwt with Flys, and Fwt-GCN with Flys-GCN), in agreement with these proteins not being cleaved. When the same F protein preparations were subjected to SDS-PAGE in the absence of reducing agents, the migration of the non-cleaved F proteins did not appear to be much affected (Fig. 2). In contrast, while the Fwt and Fwt-GCN proteins clearly ran at a lower position in the gel than the Flys and Flys-GCN proteins under reducing conditions, the difference in the electrophorectic mobility appeared much smaller in the absence of reducing agents, in agreement with the F2 part still being attached to the F1 part via disulfide bridges also in the furin-cleaved proteins. The small difference in electrophoretic mobility between the cleaved and non-cleaved F proteins that was still noticeable is most likely explained by the dissociation of the glycosylated p27 sequence [18] from the cleaved proteins. Interestingly, the electrophoretic mobility of the cleaved F proteins was dramatically changed when the preparations were not heated prior to electrophoresis under non-reducing conditions. In contrast to the non-cleaved proteins (Flys and Flys-GCN), the majority of the Fwt and Fwt-GCN proteins migrated at a much higher position in the gel in the absence of heating. The appearance of these higher order F protein structures may be explained by the cleaved F proteins adopting a stable postfusion conformation, which is characterized by the presence of an extremely stable 6HB [11], [12] and which is resistant to SDS unless the preparations are heated. This interpretation is in agreement with previous studies [18], [19], [20], [37], [38], which showed that the large majority of the soluble, cleaved F ectodomains adopts a postfusion conformation. Strikingly, the putative postfusion conformation is not prevented when the ectodomain is extended with an artificial trimerization domain. The stable postfusion form of F is not formed, however, when the F proteins are not cleaved.

**Figure 3:**
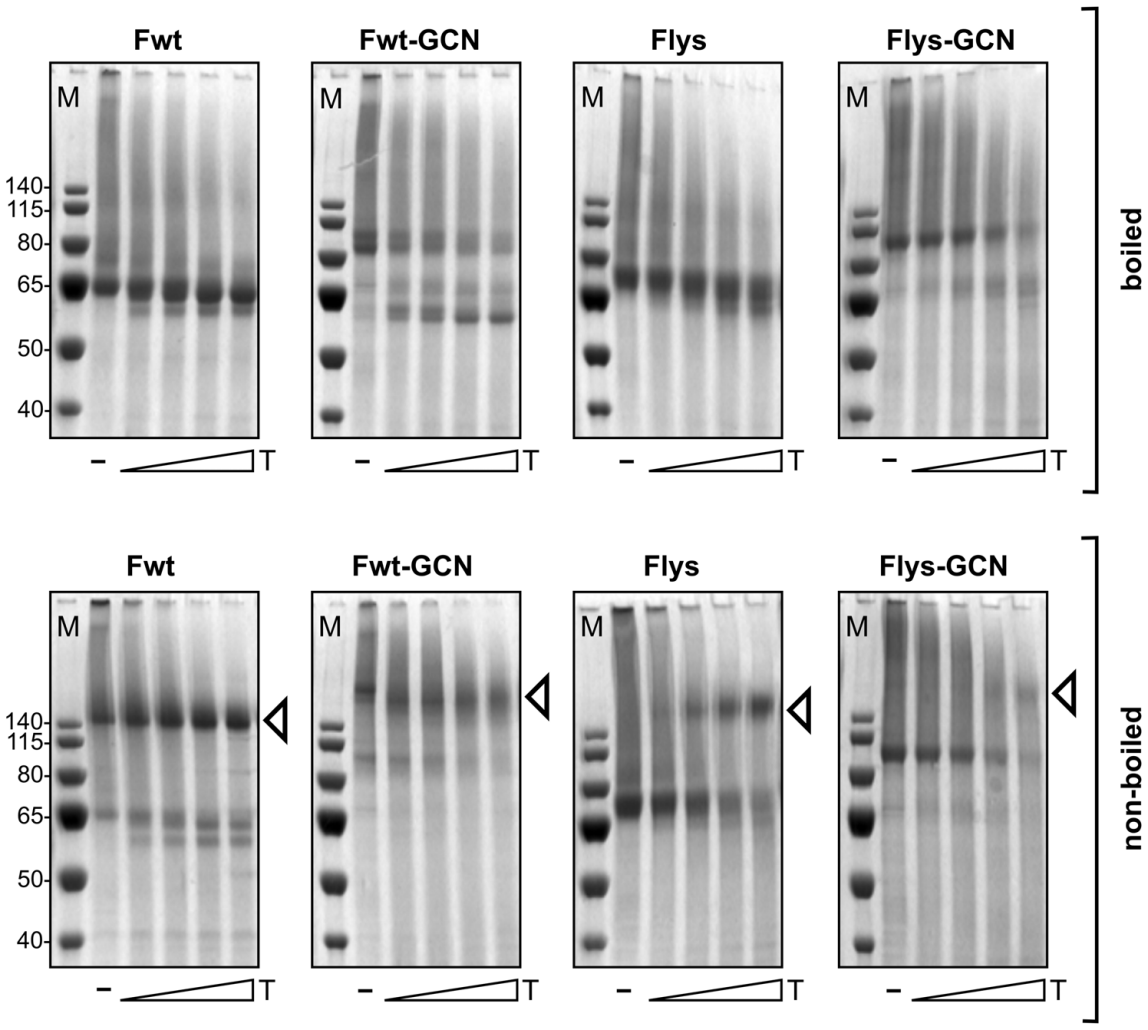
Trypsin proteolysis of recombinant F proteins. Purified F proteins were mock treated (−) or treated with increasing amounts of TPCK-Trypsin (T; increasing amounts indicated by the triangles). F proteins were subsequently analyzed by SDS-PAGE using non-reducing conditions, with (top panels) or without (bottom panels) prior boiling of the samples and stained using colloidal blue. Fwt-GCN and Flys-GCN contain a C-terminal LysM domain and ST3 tag. Fwt and Flys contain a C-terminal ST3 tag. The size of the molecular mass markers (in kDa) is shown on the left of each panel (M). Arrowheads indicate the positions in the gel of the SDS-resistant higher-order structures that are only observed when the samples were not heated prior to electrophoresis.

To confirm and extend these observations, we subsequently performed an experiment in which the purified F proteins were subjected to limiting proteolysis followed by SDS-PAGE under non-reducing conditions. As the furin-cleavage sites in Flys and Flys-GCN had been mutated by substitution of the arginines by lysines, these positions in F are still sensitive to trypsin digestion. Treatment of Flys and Flys-GCN proteins with trypsin will thus result in cleavage of these proteins and possibly in formation of the SDS-resistant higher-order structure presumably corresponding to the F protein postfusion conformation (Fig. 3). Fwt and Fwt-GCN proteins were included as controls. The purified proteins were detected using Colloidal Blue staining. Again, Fwt and Fwt-GCN ran at their expected positions in the gel, when the samples were heated prior to electrophoresis. When the samples were not heated, proteins ran at a much higher position in the gel in agreement with the results shown in Fig. 2. As expected, treatment of these samples with trypsin did not affect the migration of the higher order structures much. The higher order structures for Fwt-GCN appeared somewhat less distinct, which may be related to the trypsin treatment resulting in the removal of the F protein tags to some extent as demonstrated by the appearance of lower migrating F protein species when the samples were heated prior to electrophoresis (Fig. 3) and by Western blot analysis using tag-specific antibodies (data not shown). Treatment of the non-cleaved F proteins (Flys and Flys-GCN) with trypsin resulted in the appearance of F proteins migrating at a much higher position in the gel under non-reducing conditions, at least when the samples were not boiled prior to electrophoresis, similarly to their Fwt and Fwt-GCN counterparts. The formation of the SDS-resistant higher-order structures was more apparent for Flys than for Flys-GCN, indicative of the formation of the putative postfusion conformation being impaired to some extent by the GCN4 trimerization motif.

**Figure 4:**
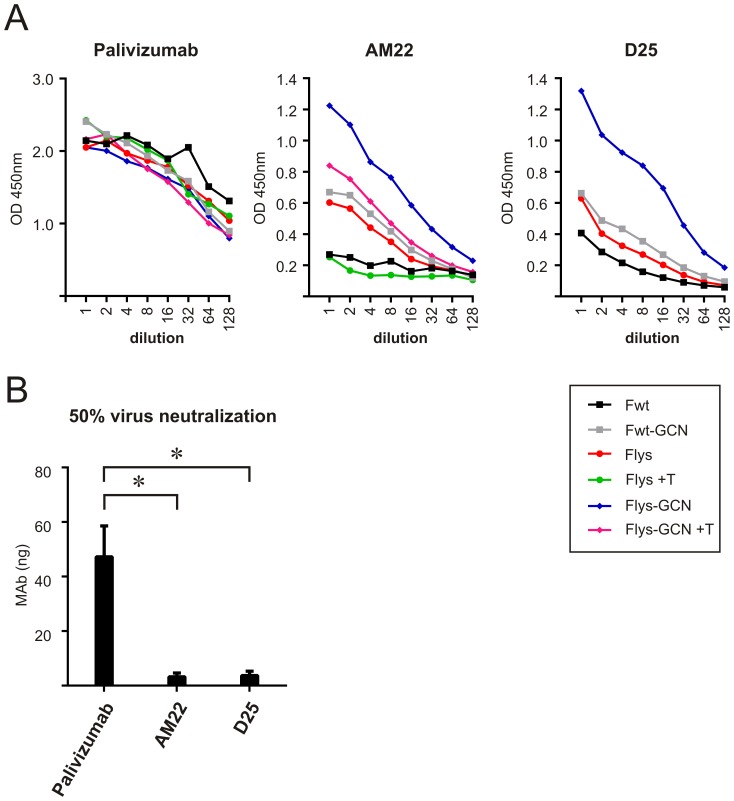
Reactivity of recombinant F proteins with neutralizing antibodies. A) ELISA analysis of recombinant F proteins. Purified F proteins were coated on 96-well plates; when indicated samples were treated with TPCK trypsin (+ T) prior to coating. The reactivity of the recombinant proteins with different neutralizing MAbs was analyzed by applying 2-fold serial dilutions of Palivizumab (starting with 0.375 µg/ml), AM22 (starting with 3.5 µg/ml) or D25 (starting with 5 µg/ml). Binding of the antibodies was detected using HRP-conjugated secondary antibodies. Fwt-GCN and Flys-GCN contain a C-terminal LysM domain and ST3 tag. Fwt and Flys contain a C-terminal ST3 tag. B) Neutralization of RSV by MAbs. The amount needed of each MAb to achieve 50% neutralization of virus infectivity is graphed. The error bars indicate the standard deviations (* P<0.05 in Student’s t test).

Next, we probed the reactivity of our F protein preparations with RSV neutralizing MAbs Palivizumab, AM22 and D25 using an ELISA format. While Palivizumab recognized the F protein in its postfusion conformation [19], [20], it also appears to recognize other conformational states, which would explain its neutralizing activity [45]. MAbs AM22 and D25, although specific for RSV F, were previously shown to display very little reactivity to cleaved, soluble F protein ectodomains, which likely adopt a postfusion conformation [35]. In agreement herewith, these antibodies were recently identified as being prefusion-specific [46]. As shown in Fig. 4A, Palivizumab displayed a concentration-dependent binding to all F protein preparations, consistent with this antibody recognizing the F protein irrespective of its conformational state. In contrast, AM22 was not able to bind Fwt, in agreement with the conclusion that this protein adopts a postfusion conformation. However, intermediate binding was observed when the cleaved protein was extended with the trimerization motif (Fwt-GCN) or when cleavage was prevented (Flys). The highest reactivity was observed when these two features were combined (Flys-GCN). Trypsin treatment of Flys and Flys-GCN prior to coating of the wells resulted in reduced AM22 reactivity, becoming comparable to the reactivity observed with the cleaved F proteins (Fwt and Fwt-GCN). Reactivity of Palivizumab with the F proteins was not affected by the trypsin treatment. The reactivity of D25 with the different F protein preparations was similar to that of AM22. When we compared the ability of the different MAbs to neutralize RSV infection of HEp-2 cells, much less AM22 or D25 than Palivizumab was needed to achieve 50% neutralization (Fig. 4B). In conclusion, our results indicate that binding of AM22 and D25, but not Palivizumab, differs between the different F protein preparations. Fwt, which presumably adopts the postfusion conformation, is least detected by AM22 and D25, while the highest reactivity was observed for Flys-GCN. Apparently, Flys-GCN displays one or more additional epitopes recognized by neutralizing antibodies than do F protein ectodomains that adopt the postfusion conformation. Importantly, AM22 and D25 that recognize these additional epitopes are very potent inhibitors of RSV infection.

### Binding of Recombinant Soluble RSV F Proteins to BLPs

**Figure 5:**
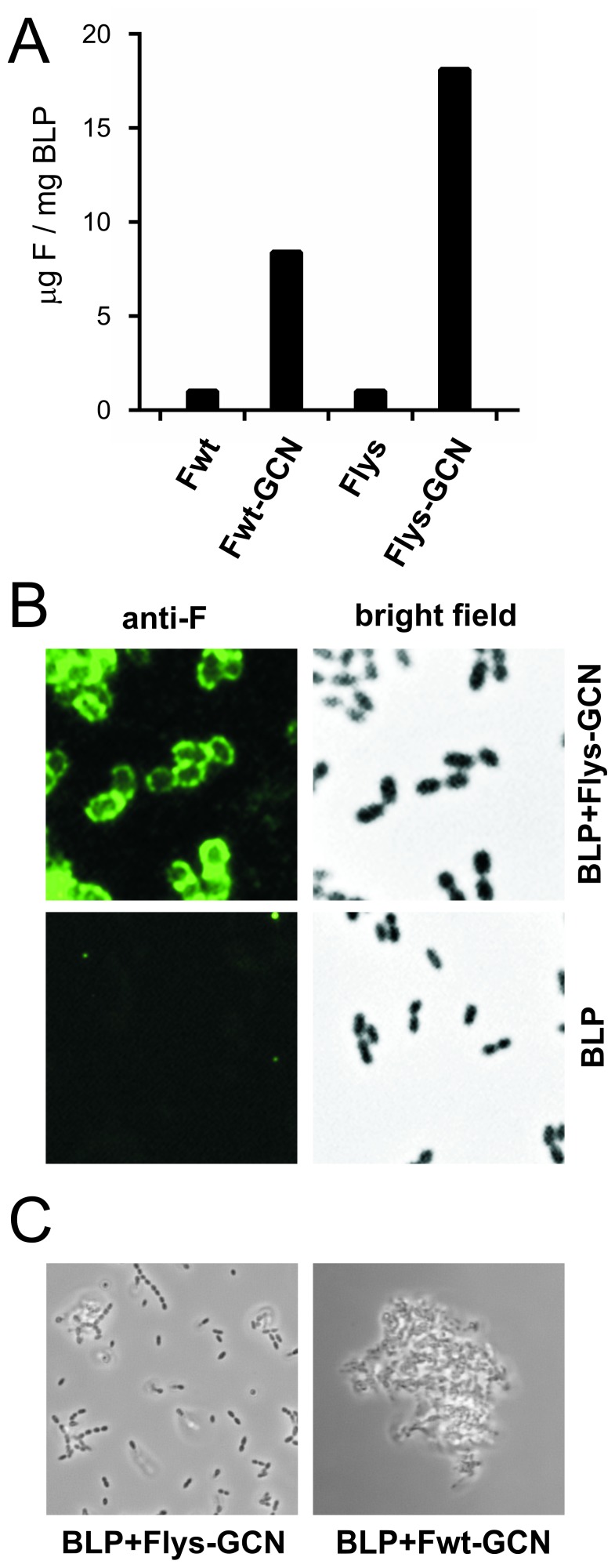
Binding of recombinant F proteins to BLPs. A) Binding efficacy of the various F proteins (Fwt, Fwt-GCN, Flys, and Flys-GCN; all carrying a C-terminal LysM domain, but no ST3 tag) to BLPs was determined by incubation of an equal amount (28 µg) of the F proteins with 1 mg of BLPs using standard conditions. The amount of F protein bound to the BLPs was determined by comparative SDS-PAGE analysis in which Colloidal Blue staining of F proteins was compared with that of BSA standards. B) To analyze the binding pattern of F to BLPs, BLPs carrying Flys-GCN with a LysM domain (BLP-F) were generated using standard conditions followed by incubation with Palivizumab and FITC-labeled goat-anti-human secondary antibodies. As a control “empty” BLPs were used. The preparations were analyzed using bright field and fluorescence microscopy. C) BLPs displaying Fwt-GCN or Flys-GCN were analyzed using bright field microscopy.

As we aim to develop a needle-free, mucosal vaccine based on BLP technology, we next assessed the binding of LysM domain-containing F proteins to the BLPs. To this end, culture media of cells that had been transfected earlier with plasmids encoding the different LysM-tagged F ectodomains, were incubated with the BLPs, after which the BLPs were collected by centrifugation. The amount of F protein bound to the BLPs was determined by comparative Colloidal Blue staining of F proteins in SDS-PA gel relative to BSA standards (Fig. 5A). While no binding was detected in the absence of a LysM domain (data not shown), LysM domain-mediated binding of F to the BLPs was much more efficient in the presence of the GCN4 trimerization domain. Apparently, the trimerization domain somehow enhances binding of F proteins that carry a single LysM domain to the BLPs. The binding of F to the BLPs was also visualized by confocal microscopy using Palivizumab (Fig. 5B), showing an even distribution of F at the surface of the BLPs. Finally, when analyzing the BLPs loaded with F we noticed that, in contrast to the BLPs carrying Flys-GCN, the BLPs displaying Fwt-GCN aggregated (Fig. 5C). This result is in agreement with the Fwt-GCN protein -unlike Flys-GCN- adopting (at least in part) a postfusion conformation whereby the hydrophobic fusion peptide becomes exposed, which subsequently results in aggregation of the F protein-bound BLPs.

### Immunization of Mice

**Figure 6:**
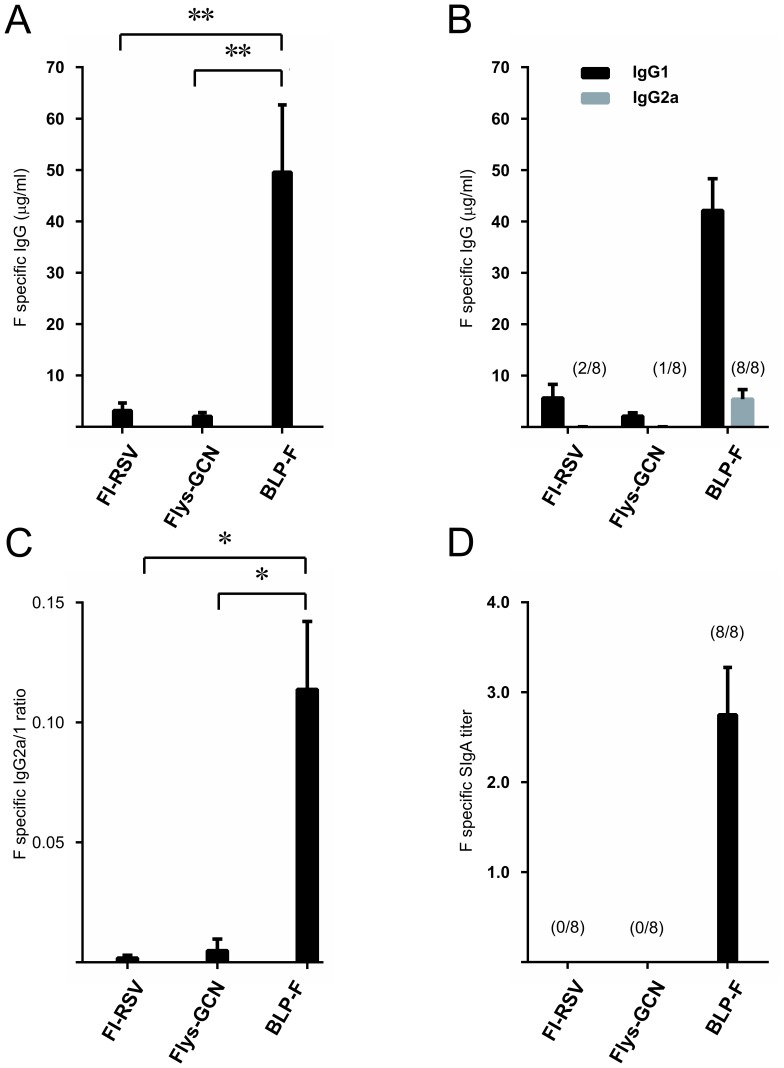
Immunization studies. Mice were immunized three times either intranasally with BLP-F or with unadjuvanted Flys-GCN4, or intramuscularly with FI-RSV with 14 day intervals. Samples were taken 28 days after the last vaccination. A) RSV-F specific IgG titers. B) RSV-F specific IgG1 and IgG2a titers. C) ratio of RSV-F specific IgG2a and IgG1 titers. D) RSV-F specific SIgA titers in the nose. Standard error of the mean (SEM) is indicated by the error bars. Numbers above the bars indicate the number of responders per group (* P≤0.05; ** P≤0.01 in Mann-Whitney U test).

Because Flys-GCN displays a more extensive and relevant epitope repertoire than postfusion F, and does not cause BLPs to aggregate upon binding (in contrast to BLPs loaded with Fwt-GCN), we performed immunization experiments in mice with BLPs displaying Flys-GCN (referred to as BLP-F). First, we studied the antibody response after three intranasal immunizations with such BLP preparations. For comparison, mice were also immunized three times intranasally with unadjuvanted Flys-GCN protein or intramuscularly with FI-RSV. All vaccinated animals displayed F-specific IgG levels, with the highest levels (15–24 fold higher) being observed for the animals vaccinated with BLP-F (Fig. 6A), while no such antibodies were observed after immunization with “empty” BLPs (data not shown). When characterizing the phenotype of the immune responses we found that mice immunized with BLP-F displayed higher F-specific IgG2a levels than animals that had received FI-RSV (Fig. 6B) or unadjuvanted Flys-GCN protein, resulting in a significantly higher IgG2a/IgG1 ratio (Fig. 6C; P≤0.05). These observations indicate that the antibody response induced by intranasal application of BLP-F displays a more balanced Th1/Th2 phenotype than the response induced by FI-RSV, which is severely Th2-skewed as expected [47], [48], [49]. Furthermore, F-specific SIgA titers in the nose were only observed after intranasal immunization with BLP-F and not with FI-RSV or unadjuvanted Flys-GCN protein (Fig. 6D), indicating that only BLP-F is capable of inducing mucosal immune responses in the respiratory tract.

**Figure 7:**
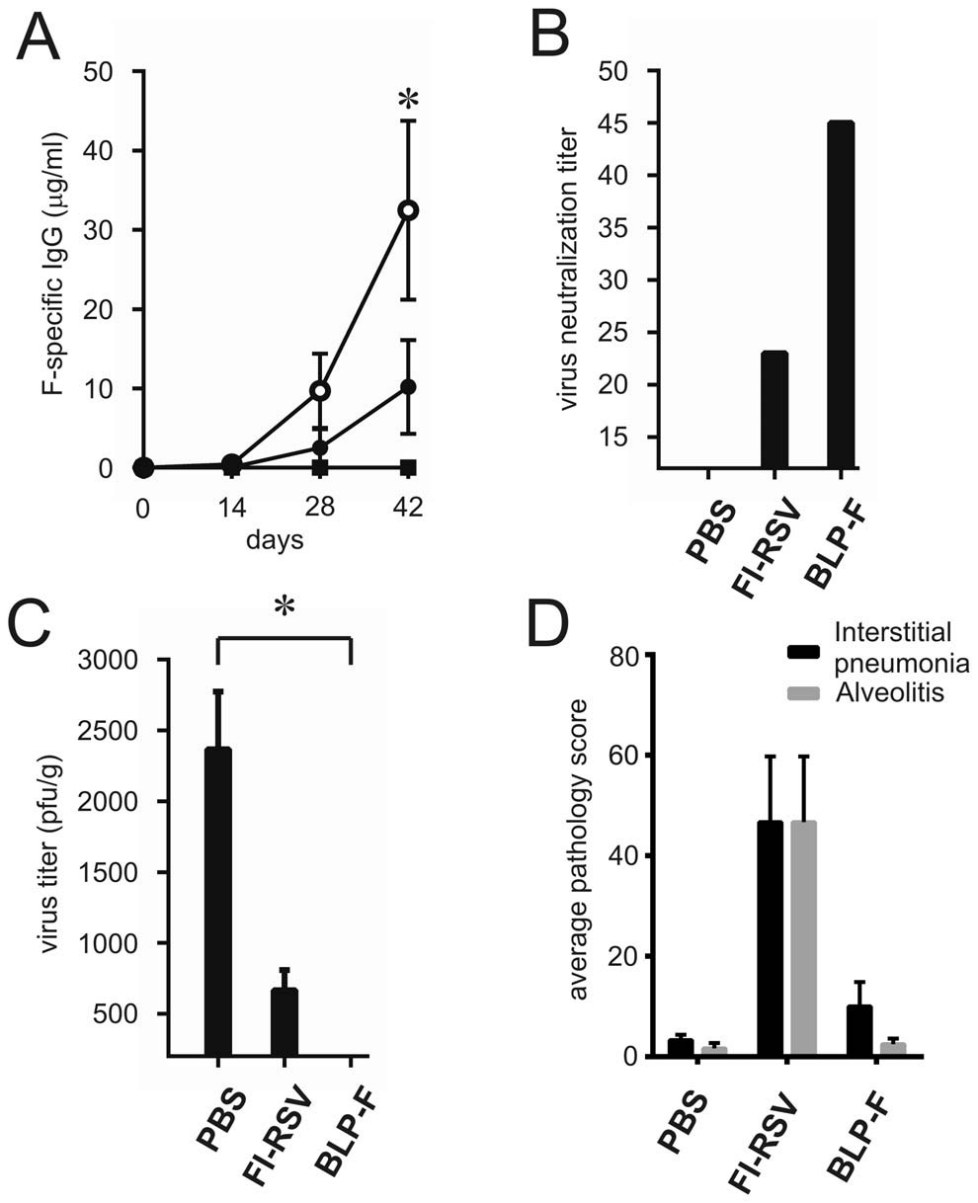
Immunization and challenge studies in mice. Mice were (mock-)vaccinated three times either intranasally with BLP-F (○) or PBS (▪) or intramuscularly with FI-RSV (•) with 14 day intervals followed by a challenge with RSV/A/long (10^6^ pfu) at 14 days after the last vaccination. A) F-specific IgG titers before immunization (day 0) and 2 weeks after each immunization (days 14, 28 and 42). B) RSV neutralization titers after three immunizations (serum pool of all animals of each group, day 42). C) Virus titers in the lungs at 5 days after challenge. The limit of detection is 200 pfu/gr. D) 5 days post challenge the lungs were harvested for pulmonary histopathology examination. Interstitial pneumonia and alveolitis were scored as described in the Materials and Methods. Standard error of the mean (SEM) is indicated by the error bars. The group receiving BLP-F was compared with the other groups on day 42 using a Mann-Whitney U test (* P≤0.05 ).

Next, we determined whether BLP-F is able to induce protective immunity against an RSV infection. To this end, mice were vaccinated three times intranasally with BLP-F; animals (mock-)vaccinated with PBS or FI-RSV (intramuscularly) were included as controls. Fourteen days after the last vaccination the mice were challenged by inoculation with RSV/A/Long (10^6^ pfu). Serum samples collected at days 28 and 42 showed that all vaccinated animals, except the ones that received PBS, displayed F-specific IgG levels, with the highest levels being observed for the animals vaccinated with BLP-F (Fig. 7A; significant at day 41, P≤0.05). These results are in agreement with the virus neutralization titers determined in pooled sera collected at day 42 (Fig. 7B). The viral infectivity titers in the lungs were determined at 5 days post inoculation. The highest titers were observed in the mock-vaccinated animals that received PBS, while no titers above the detection limit were observed after vaccination with BLP-F (Fig. 7C). The results indicate that BLP-F given intranasally is capable of inducing protective immunity in mice. Moreover, pulmonary histopathology examination showed no scores of interstitial pneumonia and alveolitis in the mice vaccinated with BLP-F, whereas as expected high levels were scored in lungs of animals vaccinated with FI-RSV (Fig. 7D).

### Vaccination of Cotton Rats

**Figure 8:**
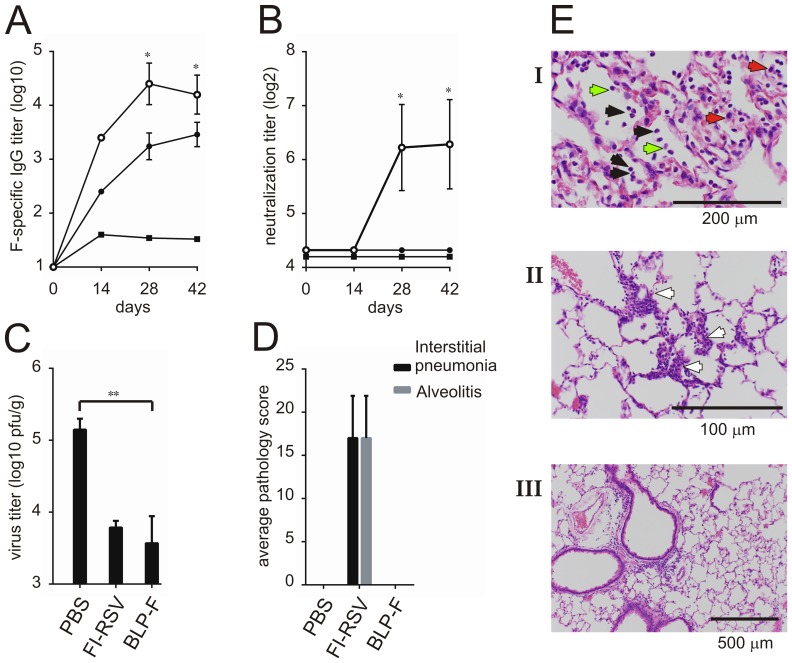
Immunization, challenge studies and lung pathology post challenge in cotton rats. Cotton rats (N = 5) were (mock-)vaccinated three times (starting at day 0) with either intranasally with BLP-F (○), PBS (▪) or intramuscularly with FI-RSV (•) with 14 day intervals followed by a challenge with RSV/A/long (10^6^ pfu) at 14 days after the last vaccination. A) Serum IgG titers after 0 (day 0), 1 (14 days), 2 (28 days) and 3 (42 days) immunizations. B) RSV neutralization titers in cotton rat sera before (day 0) and 2 weeks after each immunization (days 14, 28, 42). C) Virus titers in the lungs at 5 days after challenge. The limit of detection is 200 pfu/gr. D) 5 days post challenge the lungs were harvested for pulmonary histopathology examination. Interstitial pneumonia and alveolitis were scored as described in the Materials and Methods. Standard error of the mean (SEM) is indicated by the error bars. The group receiving BLP-F was compared with the other groups using a Mann-Whitney U test (* P≤0.05; ** P≤0.01). The asterisks in A and B indicate a significant difference between the group receiving BLP-F and the other two groups except for one of the asterisks in A (day 42), which only indicates a significant difference between the groups that received BLP-F and PBS. E) Examples of hematoxylin and eosin-stained lung tissues of animals vaccinated with FI-RSV (top [I] and middle [II] picture) or BLP-F (bottom picture[III]). The black, red and green arrows point at granulocytes, macrophages, and lymphocytes, respectively. The white arrow indicates the widening of the alveolar walls due to the cell infiltration (alveolitis).

Finally, a vaccination challenge experiment was performed using the cotton rat animal model of RSV. This model is particularly suitable to reproduce the symptoms of vaccination-associated enhanced disease that were observed after natural infection of children that had previously been vaccinated with FI-RSV [50], [51]. Thus, cotton rats were (mock-)vaccinated intranasally with BLP-F or with PBS, or were vaccinated intramuscularly with FI-RSV. Except for the mock-vaccinated cotton rats, all vaccinated animals displayed high IgG titers (from day 14 post vaccination onwards), with the titers elicited by the intranasally applied BLP-F being the highest (Fig. 8A). Only the animals vaccinated with BLP-F (day 28 and 42 post vaccination) demonstrated detectable and high virus neutralization titers (Fig. 8B). The virus neutralization titers plateaued after the first boost prime-boost vaccination and no contribution of a second boost was observed. Fourteen days after the last vaccination, the cotton rats were challenged with RSV/A/Long (10^6^ pfu). At day 5 post challenge, the animals were euthanized and viral titers in the lungs were determined. Animals vaccinated with BLP-F or with FI-RSV displayed a significant reduction in lung titers (1.7 and 1.4 log10, respectively; Fig. 8C). In agreement with our observations in mice, pulmonary histopathology examination showed no scores of interstitial pneumonia and alveolitis in the cotton rats vaccinated with BLP-F, whereas as expected high levels were scored in lungs of animals vaccinated with FI-RSV (Fig. 8D and E). In conclusion, we show that vaccination of both mice and cotton rats with BLP-F results in protective immunity, which is not associated with vaccination-related enhanced disease symptoms.

## Discussion

Globally, RSV is the most common cause of childhood acute lower respiratory infections (ALRI) and a major cause of hospital admission due to severe ALRI [52]. In the elderly, hospitalization rates for RSV are similar to those associated with influenza [53]. Despite the morbidity and mortality caused by RSV, a licensed vaccine against RSV is not available. Here we show recombinant soluble F protein, when mutated in its furin cleavage sites and C-terminally extended with a trimerization motif, to assume a prefusion-like conformation rather than adopting the postfusion form, and to expose a more extensive and relevant epitope repertoire. Intranasal vaccination of mice or cotton rats with BLPs loaded with these recombinant F proteins (BLP-F) induced high RSV F-specific IgG and SIgA levels and virus neutralizing antibody titers and significantly decreased RSV titers in the lung upon RSV challenge. In contrast to vaccination with FI-RSV, immunization with BLP-F induced no enhanced respiratory disease symptoms after infection with a high dose of RSV.

Different recombinant soluble F protein preparations were analyzed in this study. Soluble, cleaved F protein (Fwt) was shown to adopt a higher-order structure that was resistant to 2% SDS, indicative of the formation of the very stable 6HB, and to display high reactivity with MAb Palivizumab, which recognizes both pre- and postfusion forms of F [19], [20], [45], but not with the prefusion-specific MAbs AM22 and D25 [35], . Collectively, these results indicate that the soluble, cleaved F protein is in the postfusion form, which is in agreement with observations made in several other studies where similar soluble F protein constructs were expressed [18], [19], [20], [37], [38]. In one study, the majority of the soluble, cleaved F protein was in the prefusion state [39], but this conformation was not stable and the protein readily adopted the postfusion state by exposure to low molarity buffer.

Interestingly, addition of an artificial trimerization motif to the carboxy terminus of the soluble cleaved F protein (Fwt-GCN) did not prevent the F protein from adopting the postfusion conformation, as judged by the formation of the SDS-resistant, heat-sensitive, higher-order structure. Consistently, BLPs carrying Fwt-GCN were found to aggregate, which can be attributed to the hydrophobic interactions between fusion peptides that are exposed in the postfusion conformation. The somewhat increased reactivity of Fwt-GCN with AM22 and D25 when compared to Fwt is probably explained by a proportion of this protein not adopting the postfusion conformation. Actually, the ability of the majority of Fwt-GCN to adopt the postfusion conformation is remarkable as the addition of the GCN4 trimerization motif to several other class I fusion proteins was sufficient to keep these proteins in the prefusion conformation [32], [40], [41]. While heating of the GCN4-extended, soluble, cleaved form of the F protein of parainfluenza virus (PIV) 5, which is also a paramyxovirus, was required for this protein to adopt the postfusion conformation [54],heating is not required for its RSV F counterpart (Fwt-GCN; this study). These results thus indicate that the metastable prefusion conformation of the RSV F protein is intrinsically more unstable than that of other class I fusion proteins.

Preventing furin-cleavage of the soluble RSV F ectodomain clearly affected the conformational state of the secreted proteins. Non-cleaved soluble F proteins did not adopt the postfusion conformation as judged from the absence of SDS-resistant, heat sensitive, higher-order structures. Non-cleaved F proteins were also somewhat better recognized by the MAbs AM22 and D25 than their cleaved counterparts. Trypsin digestion of the cleavage mutant F proteins resulted again in SDS-resistant, heat sensitive, higher order structures with concomitant reduction in reactivity with AM22. Binding of non-cleaved F proteins to BLPs did not result in aggregation of the BLPs, indicating that the fusion peptide was not exposed. These results are in agreement with other observations showing that non-cleaved RSV F adopts another conformational state than cleaved F, exemplified by different F protein morphologies as observed by electron microscopy [18], [37], [38]. Furthermore, non-cleaved F proteins do not form rosettes that result from hydrophobic interactions between the fusion peptides as is the case for the cleaved F [18], [37], [38]. Strikingly, however, the F proteins of two other paramyxoviruses (PIV3 and PIV5) were shown to adopt a postfusion conformation even in the absence of F protein cleavage [54], [55], although for PIV5 this non-cleaved, heated F protein was found not to be recognized by an antibody that specifically recognizes the (cleaved) postfusion state [54].

Of the different F proteins produced in this study the Flys-GCN construct came out as the most attractive antigen for vaccinations. Flys-GCN, the non-cleaved F protein that is carboxy-terminally extended with the GCN4 trimerization motif, did not adopt the putative postfusion conformation and displayed the highest reactivity with prefusion-specific MAbs AM22 and D25 [46], indicative of the protein assuming a prefusion-like conformation. More research is needed to elucidate whether the Flys-GCN protein adopts the native prefusion state or rather an intermediate conformation. Importantly, the AM22 and D25 antibodies have a much higher anti-RSV neutralizing capacity than Palivizumab ([35], [46] and this study). As an antigen that mimics a prefusion-like form of F and exposes at least one additional epitope recognized by neutralizing antibodies compared to the postfusion form, Flys-GCN was bound to BLPs to generate the BLP-F vaccine candidate. A direct comparison with the other, postfusion F protein preparations was not pursued because of the unacceptable aggregation that we observed for the BLPs displaying Fwt-GCN.

Intranasal vaccination of mice and cotton rats with BLP-F resulted in the induction of F-specific IgG levels in sera and SIgA titers in nose lavages that were higher than those observed in animals vaccinated intramuscularly with FI-RSV or intranasally with unadjuvanted F protein. Moreover, high serum levels of virus neutralizing antibodies were only observed after vaccination with BLP-F, both in cotton rats and mice. These results confirm the known adjuvanting properties of the BLPs when used intranasally [22], [23] and show that intranasal application of BLP-F results in RSV F-specific systemic and mucosal immune responses. Virus neutralizing antibodies directed against the RSV F protein are known to correlate with protection against RSV infection. This is demonstrated amongst others by the successful, and currently only available immune treatment of RSV infection with Palivizumab, which prevents serious RSV illness in premature children and in patients with bronchopulmonary dysplasia [56]. Consistently, the high levels of virus neutralizing serum antibodies and SIgA titers in nose lavages elicited in this study by intranasal vaccination with BLP-F resulted in protection both in mice and cotton rats as evidenced by the significantly reduced virus titers in the lungs of these animals.

Whether local F-specific SIgA is important for protection against RSV infection and disease is not well established. Passive nasal transfer of a mouse monoclonal IgA antibody against RSV F glycoprotein significantly reduced viral shedding in the nose, throat, and lungs in a rhesus monkey model of RSV infection suggesting a role for local SIgA in protection [57]. Whether the BLP-F induced local SIgA responses contribute to protection cannot be deduced from the present set of results. However, in previous experiments intranasal administered influenza vaccine mixed with BLPs resulted in a superior protection against homologous and heterologous influenza virus infection compared to conventional intramuscular immunization, even though hemagglutination inhibition titers of sera derived from animals that received the intranasal BLP vaccine were not elevated compared to the other vaccinated animals. These results suggest a role of local immunity in the protective capacity of intranasal BLP-based vaccines [24]. Clearly, it will be of interest to study whether BLP-F is capable of inducing a similar first line of defense against RSV infection at the upper and/or lower respiratory tract mucosa.

Our results indicate that vaccination with BLP-F was safe when used in both mice and cotton rats. No signs of enhanced respiratory disease symptoms in the form of alveolitis or interstitial pneumonia were observed in the lungs of BLP-F vaccinated animals that were subsequently challenged with RSV. In contrast, vaccination with FI-RSV followed by RSV challenge induced severe enhanced disease symptoms as expected and observed before [51]. The exact mechanism(s) by which vaccination with FI-RSV induces enhanced respiratory disease in naïve infants after infection is still not clear. Recent work in mice implicated the failure of FI-RSV in inducing virus neutralizing antibodies to poor affinity maturation, likely caused by the lack of sufficient TLR stimulation [58], as well as to an excessively Th2-skewed immune response [49], [59]. The absence of enhanced disease symptoms after vaccination with BLP-F may therefore be explained by the BLP-F driven induction of robust serum levels of virus neutralizing antibodies accompanied by a relatively high IgG2a/IgG1 ratio, which is typical for a better balanced Th1/Th2 type immune response, compared to animals that received FI-RSV. Although the phenotype of the immune response induced by nasal administration of BLP-F needs further exploration, the type of response is in agreement with previous studies using BLP-based vaccines. For example, intranasal immunization with BLPs mixed with influenza subunit vaccine elicited Th1 skewed immune responses compared to those induced by intramuscular and intranasal administered influenza subunit vaccine alone [23]. With respect to the TLR stimulation mediated affinity-maturation it is noteworthy that BLPs act as a TLR-2 agonist driving the maturation of human dendritic cells and macrophages in vitro [22], while the role of TLR-2 in BLP dependent immune stimulation was recently confirmed by in vivo experiments using TLR2^−/−^ mice (unpublished data).

In conclusion, we have shown that at least one epitopes recognized by neutralizing antibodies that are not present in RSV F proteins that adopt a postfusion conformation can be preserved in recombinant soluble RSV F proteins by mutation of the furin-cleavage sites in combination with the addition of a C-terminal trimerization motif. Intranasal vaccination of mice and cotton rats with BLP-based vaccines carrying these prefusion-like F proteins was shown to result in high levels of F-specific IgG in sera, local SIgA in the nose and in virus neutralizing antibodies. Mice and cotton rats vaccinated with this BLP-F showed reduced virus titers in the lungs after challenge, in the absence of enhanced disease symptoms, in contrast to animals that received FI-RSV.

## Supporting Information

Figure S1 **Sequences of the GCN4 trimerization domain and the tags used.** The carboxy-terminal residues of the RSV F protein ectodomain are shown in blue. The triple Strep-tagII (ST3; italic, green), GCN4 trimerization domain (red, bold) and the LysM domain (underlined, including LysM linker sequences) are indicated. F proteins containing ST3 were used in the *in vitro* analysis, while the LysM domain-containing proteins lacking the ST3 tag were used for BLP binding.
(DOCX)
