# Supplementary material for: A Protective and Safe Intranasal RSV Vaccine Based on a Recombinant Prefusion-Like Form of the F Protein Bound to Bacterium-Like Particles
Source: PLoS One. 2013 Aug 12;8(8):e71072. doi: 10.1371/journal.pone.0071072 (PMC3741363; doi:10.1371/journal.pone.0071072)
Supplement: Figure S1 — Sequences of the GCN4 trimerization domain and the tags used. The carboxy-terminal residues of the RSV F protein ectodomain are shown in blue. The triple Strep-tagII (ST3; italic, green), GCN4 trimerization domain (red, bold) and the LysM domain (underlined, including LysM linker sequences) are indicated. F proteins containing ST3 were used in the in vitro analysis, while the LysM domain-containing proteins lacking the ST3 tag were used for BLP binding. (DOCX) [file pone.0071072.s001.docx]

**ST3**

RKSDELLHNLIN*DYKDDDDKAGPGWSHPQFEKGGGSGGGSGGGSWSHPQFEKGGGSGGGSGGGSWSHPQFEK*

**GCN4-LysM-ST3**

RKSDELLHNLIK**RMKQIEDKIEEIESKQKKIENEIARIKK**GNTNSGGSTTTITNNNSGTNSSSTTYTVKSGDTLWGISQR

YGISVAQIQSANNLKSTIIYIGQKLVLTGSASSTNSGGSNNSASTTPTTSVTPAKPTSQTT*DYKDDDDKAGPGWSHPQFE*

*KGGGSGGGSGGGSWSHPQFEKGGGSGGGSGGGSWSHPQFEK*

**LysM**

RKSDELLHNLIKGNTNSGGSTTTITNNNSGTNSSSTTYTVKSGDTLWGISQRYGISVAQIQSANNLKSTIIYIGQKLVLT

GSASSTNSGGSNNSASTTPTTSVTPAKPTSQTT

**GCN4-LysM**

**RKSDELLHNLIKRMKQIEDKIEEIESKQKKIENEIARIKKGNTNSGGSTTTITNNNSGTNSSSTTYTVKSGDTLWGISQR**

**YGISVAQIQSANNLKSTIIYIGQKLVLTGSASSTNSGGSNNSASTTPTTSVTPAKPTSQTT**
